# Molecular Friction Trumps Viscosity in Angstrofluidic Transport

**DOI:** 10.1002/smll.75044

**Published:** 2026-08-04

**Authors:** Jaber Al Hossain, Murat Barisik, Chirodeep Bakli, Suman Chakraborty, BoHung Kim

**Affiliations:** ^1^ School of Mechanical Engineering University of Ulsan Ulsan South Korea; ^2^ Mechanical Engineering Department University of Tennessee at Chattanooga Chattanooga Tennessee USA; ^3^ School of Energy Science and Engineering Indian Institute of Technology Kharagpur Kharagpur West Bengal India; ^4^ Department of Mechanical Engineering Indian Institute of Technology Kharagpur Kharagpur West Bengal India

**Keywords:** continuum breakdown, corresponding viscosity coefficient (CVC), ergodic hypothesis, single‐file molecular transport, sub‐nanometer fluid dynamics

## Abstract

In the realm of ultra‐confined nanofluidics, where channels barely accommodate a single molecule, classical continuum theories would inevitably falter. Fundamental properties like viscosity, wettability, and velocity profiles lose their conventional meaning, leaving a critical gap in our ability to predict and engineer flow at the molecular scale, despite these features continuing to be extrapolated from the underlying molecular behavior. Here, we tackle this conceptual anomaly by probing single‐file transport of liquid argon through graphene nanopores and carbon nanotubes via molecular dynamics simulations and interpreting the same from the foundational principles of statistical mechanics as against the traditional approach of continuum property extrapolation. By invoking the ergodic hypothesis, we reconstruct long‐time velocity distributions from individual atomic trajectories, enabling the definition of a corresponding viscosity coefficient (CVC) as a continuum‐referenced resistance metric rather than a conventional effective viscosity. This physically inspired mapping bridges atomistic realities with continuum surrogates, revealing how flow resistance depends on wall‐fluid interactions and confinement‐induced geometrical features. Our framework unlocks predictive design tools for sub‐nanometer fluidic devices, with significant implications for nanofiltration, ion sieving, and next‐generation semiconductor processing, by providing a new lens to translate single‐molecule chaos into actionable engineering insight.

## Graphical abstract

Single‐file transport through sub‐1 nm graphene nanopore and carbon nanotube is investigated using molecular dynamics and ergodic trajectory sampling. The study shows that confinement geometry and wall‐fluid interaction strength govern molecular mobility, allowing atomistic motion to be translated into a continuum‐based resistance coefficient for interpreting angstrom‐scale molecular transport.

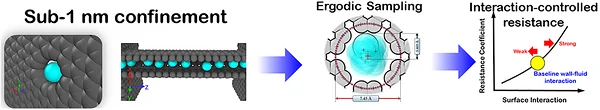

## 1 Introduction

Efficient separation and molecular‐scale transport imperatively influence technologies ranging from nanofiltration and desalination to semiconductor processing and biosensing. Recent breakthroughs in carbon‐based nanomaterials, such as graphene oxide membranes and carbon nanotubes (CNTs), have revealed ultrafast water and ion transport that defy classical continuum predictions [1, 2, 3, 4], highlighting the profound influence of channel geometry, wall chemistry, and interlayer spacing. Yet, despite these advances, the mechanisms governing resistance, velocity distribution, and energy dissipation under sub‐nanometer confinement remain largely unexplored, limiting the rational design of next‐generation fluidic devices encroaching sub‐nanometer dimensions (angstrofluidic regime).

Classical fluid mechanics treats fluids as continuous media, with smoothly varying properties such as velocity, pressure, and density, consistent with the continuum conservation equations [5, 6, 7]. While remarkably successful at macroscopic scales and even microscopic or sub‐micron scales to a considerable extent, this framework breaks down completely below nanometer scales, where molecular discreteness can potentially disrupt the flow features [8, 9, 10]. Concurrently, physics‐guided machine‐learning reparameterizations of liquid force fields recover transport‐critical properties with higher fidelity, providing a complementary bridge between molecular realism and continuum surrogates [11]. This perspective motivates our focus on how discrete, near‐wall molecular structure governs transport under extreme confinement.

In angstrofluidic channels, single‐file transport and density layering emerge, and the molecules interact distinctively with the solid walls through steric effects, surface forces, and molecular ordering [12, 13, 14, 15, 16, 17, 18], rendering the conventional concept of viscosity ambiguous. Understanding these wall‐molecule interactions is, however, critical: they control flow rates, transport efficiency, and the velocity profile, yet are inaccessible through traditional continuum approaches or even a standard extrapolation of the molecular dynamics results toward mimicking the same. These limitations render it particularly pressing in applications that demand extreme nanoscale precision. In semiconductor manufacturing, for example, fluid control within nanopores influences wafer cleaning and etching at atomic precision [19, 20], while in drug delivery and DNA sequencing, controlled single‐molecule transport is fundamental to efficacy and sensing resolution [21, 22].

In addition to confinement‐driven transport, recent micro/nanofluidic studies have demonstrated that ion transport can be actively regulated through induced‐charge electrokinetic and electroconvective mechanisms in ion‐selective systems, highlighting the broader importance of controllable transport in confined fluidic platforms [23]. Prior studies have elucidated the scale‐dependent breakdown of classical hydrodynamics. Early research in this regard demonstrated water transport rates through sub‐2 nm CNTs exceeding continuum predictions by three orders of magnitude [24]. Such findings could be linked with molecular ordering and interfacial structure to rationalize the deviations from continuum behavior [25]. Material‐specific effects, such as lower friction in CNTs compared to boron nitride, further exemplify that sub‐nanometer‐scale transport is highly geometry‐ and interfacial chemistry‐dependent [26]. The reported molecular simulations could further bring out the deviations from the classical parabolic velocity profile as the characteristic dimensions downscale to the nanofluidic regimes [27, 28, 29, 30, 31]. However, the reported investigations in this regard did not account for single‐file angstrofluidic regimes, where ensemble averaging would by itself collapse.

Here, we propose a new framework to bridge this gap, wherein, by invoking the ergodic hypothesis, the long‐time trajectory of a single molecule is used to reconstruct stable velocity profiles, capturing the transport statistics without assuming continuum‐like extrapolation. These atomistic profiles are finally mapped onto classical continuum templates (Hagen–Poiseuille for CNTs; Sampson for nanopores) by defining a configuration‐dependent “corresponding viscosity coefficient” (CVC), which quantifies interfacial, confinement‐dominated resistance. Our framework is demonstrated using molecular dynamics simulations of single‐file argon transport through graphene nanopores and cylindrical CNTs. Velocity profiles are reconstructed via ergodic averaging, interpreted with continuum templates, and used to extract the CVC, revealing how geometry and wall‐fluid interactions modulate nanoscale resistance. This methodology provides a predictive, compact, and transferable toolkit for designing angstrofluidic systems, with broad implications for nanofiltration, ion sieving, molecular sensing, and semiconductor process engineering. By linking atomistic insights to continuum surrogates, our work opens a new route for rational design and performance prediction in ultra‐confined geometries. As against attempting to predict the “real viscosity” inconsistently in such situations, our findings thus provide a practical surrogate of an effective fluidic resistance to predict flux in device‐relevant geometries, thereby translating single‐molecule chaos into engineering‐ready metrics.

## 2 Simulation Details

**FIGURE 1:**
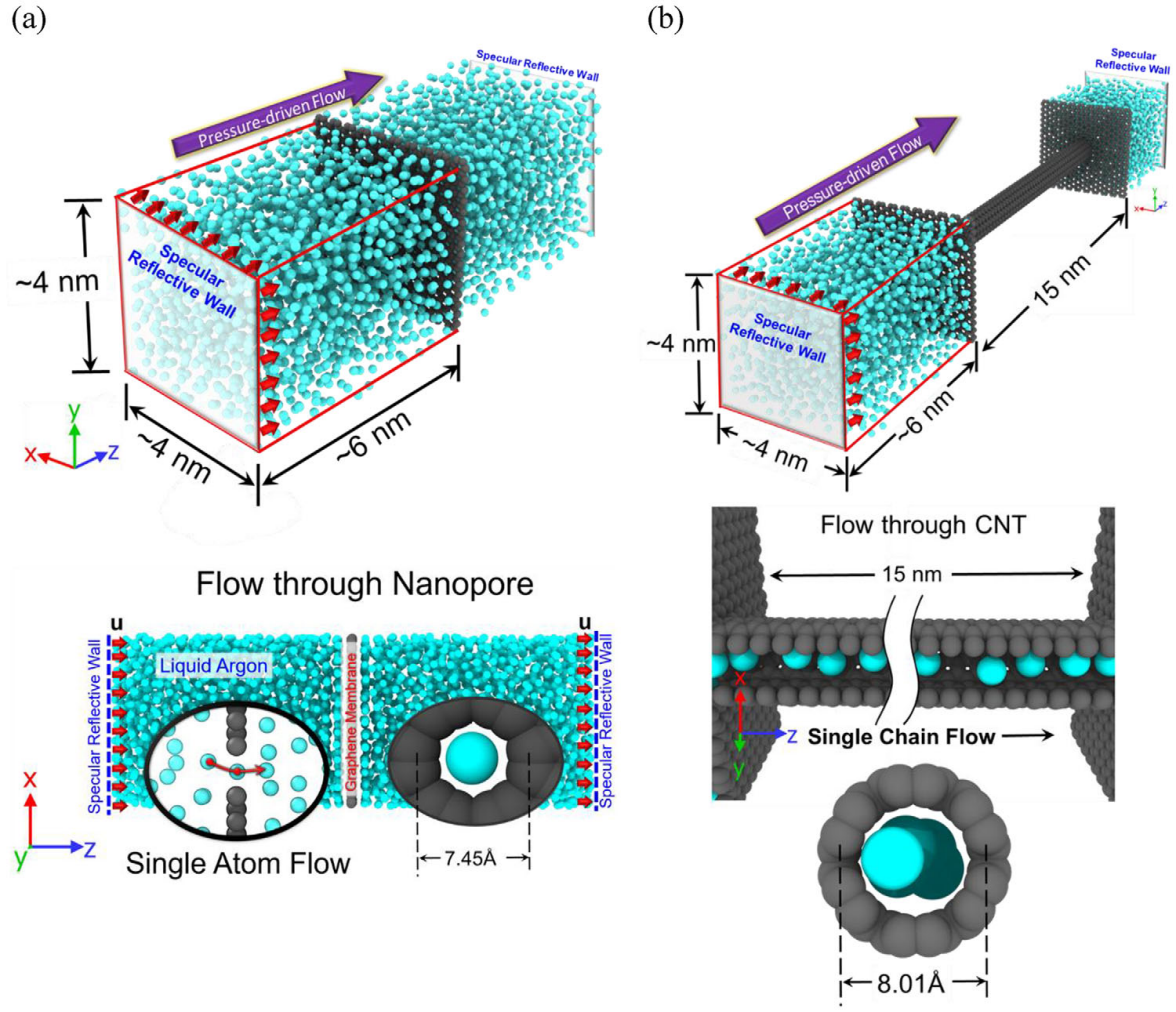
Simulation domains for single‐atom transport under angstrom‐scale confinement. (a) Nanoporous graphene membrane centered at *z* = 0 Å separating two liquid‐argon reservoirs; pore diameters tested were 5.68, 7.45, and 9.94 Å, with 7.45 Å selected for single‐file analysis. (b) Armchair CNT (8.01 Å diameter; nominal length 15 nm) aligned along z, enabling a one‐to‐one comparison between planar and cylindrical confinement at comparable aperture size. In both systems, reservoirs contain liquid Ar prepared near number density ≈ 0.8 and are driven by specular‐reflection walls.

In this study, molecular dynamics (MD) simulations were conducted to investigate fluid transport through two distinct nanofluidic systems: a nanoporous graphene membrane and a carbon nanotube (CNT). Although the simulation conditions for both systems were identical, their geometries differed, with the nanoporous membrane providing a planar passage for fluid (Figure 1a), while the CNT represented a cylindrical channel(Figure 1b). Both systems were designed to explore the behavior of liquid argon under extreme confinement, specifically focusing on single‐atom transport.

Figure 1a shows the nanopore system consisting of a graphene membrane positioned at the center of the simulation domain (z = 0 Å), effectively separating two reservoirs filled with liquid argon. The centrally located nanopore was generated by removing carbon atoms inside a circular region centered on the graphene sheet. Because of the discrete hexagonal lattice of graphene, the pore size cannot be varied continuously; instead, it increases stepwise as successive rings/groups of edge atoms are removed. Following our previous pore‐definition procedure [32]. the pore diameter was measured from the atomic centers of opposing pore‐edge carbon atoms across the pore center. Among the atomistically allowed openings, three representative pore diameters were tested: 5.68, 7.45, and 9.94 Å. The 5.68 Å pore was too narrow and allowed no argon flow, whereas the 9.94 Å pore permitted the simultaneous passage of multiple atoms, resulting in multi‐file flow. Therefore, to focus on strict single‐atom transport, the 7.45 Å pore was selected for detailed analysis because it allowed only one argon atom to pass at a time, creating the target single‐file transport condition. The system was bounded by specular reflection boundaries at z = −60 Å and z = +60 Å, ensuring confinement, while periodic boundary conditions were applied in the *x* and *y* directions, each with a length of 4 nm. The carbon atoms in the graphene membrane were fixed, providing a rigid structure for the nanopore. This rigid‐carbon approximation was adopted to isolate the effects of confinement geometry and wall‐fluid interaction strength from additional membrane deformation. Similar fixed‐carbon treatments have been used in graphene nanopore transport simulations to decouple fluid permeation from mechanical deformation of the membrane. Cohen–Tanugi and Grossman, for example, held the carbon atoms fixed in nanoporous graphene simulations specifically to decouple saltwater transport from membrane deformation phenomena [33]. Thermal wall motion, thermostatting strategy, and tube flexibility can influence interfacial momentum transfer and the absolute slip/friction magnitude in nanoconfinement [34, 35]. However, in the present work, the same rigid‐wall treatment was applied consistently to all nanopore and CNT cases; therefore, the comparative trends in velocity profile and CVC with respect to geometry and wettability remain internally consistent within the controlled model framework.

In the CNT system shown in Figure 1b, the reservoirs were separated by an armchair CNT (diameter 8.01 Å; nominal length 15 nm) aligned along the *z*‐axis. Because CNT diameter is determined by chirality and cannot be tuned continuously at the angstrom level, the nearest available armchair tube diameter, 8.01 Å, was selected to provide a comparable single‐file cylindrical confinement to the 7.45 Å graphene nanopore. The 15 nm CNT length was used to provide an extended cylindrical single‐file channel with sufficient axial sampling for the ergodic velocity reconstruction. This length choice was not intended to establish a full length‐dependent resistance map, but to enable a controlled comparison between atomically thin planar confinement and finite‐length cylindrical confinement. The CNT was positioned with one end fixed at z = 0 Å and the other at z = 150 Å, and, as in the nanopore setup, the reservoirs were filled with liquid argon with periodic boundary conditions applied in the *x* and *y* directions. This configuration allowed a direct comparison of the transport characteristics of single‐file argon flow in a cylindrical channel with those in the nanopore.

We used liquid argon modeled by the canonical Lennard–Jones (LJ) 12–6 potential as a minimal fluid to isolate confinement‐dominated transport (steric exclusion, discrete occupancy, and interfacial friction) from chemistry‐specific effects (hydrogen bonding, partial charges, or reactivity). The LJ fluid has long served as a reference system for liquids because it captures generic short‐range repulsion and dispersion with well‐defined reduced units and benchmarked properties, enabling controlled studies and reproducible comparisons across geometries and interaction strengths [36]. Using a monatomic LJ fluid yields major computational savings: we can use larger stable timesteps and avoid long‐range electrostatics. It also keeps the model minimal, with fewer parameters, so trends can be attributed cleanly to interfacial energetics and geometry rather than force‐field particulars [37, 38, 39]. Finally, the LJ/argon choice aligns with established nanofluidics and slip‐flow literature that deliberately employ simple fluids to quantify how interfacial wettability and surface corrugation control boundary conditions and flow resistance at the nanoscale, exactly the regime targeted here [8, 40].

The use of liquid argon is therefore intended as a first‐principles baseline rather than a direct representation of all practical nanofluidic liquids. Because the mechanism of single‐atom transport in angstrom‐scale confinements is not yet fully established, a monatomic LJ liquid provides a controlled starting point in which the basic effects of confinement geometry, steric exclusion, discrete occupancy, and wall‐fluid friction can be separated from additional fluid‐specific molecular interactions. For structured or hydrogen‐bonded liquids such as water, and for electrolytes, the same confinement may introduce additional resistance contributions from hydrogen‐bond rearrangement, dipolar orientation, hydration‐shell deformation, ion‐wall electrostatics, and surface charge. Thus, the present LJ system should be interpreted as a chemically minimal reference case, while future extensions to SPC/E‐type water and electrolyte models would allow the separate effects of polarity, hydrogen bonding, and Coulombic interactions to be incorporated systematically.

The interactions between argon atoms in both systems were modeled using the truncated Lennard–Jones (LJ) (12‐6) potential:

(1) $$\begin{array}{ccc} & & V_{truncated}\;\;\left(r_{ij}\right)\\ & = & 4\varepsilon \left\{\left[{\left(\frac{\sigma }{r_{ij}}\right)}^{12}-{\left(\frac{\sigma }{r_{ij}}\right)}^{6}\right]-\left[{\left(\frac{\sigma }{r_{c}}\right)}^{12}-{\left(\frac{\sigma }{r_{c}}\right)}^{6}\right]\right\} \end{array}$$

where ε represents the depth of the potential well, *r_ij_* is the distance between two interacting atoms, σ is the finite distance at which the potential is zero, and *r_c_* is the cutoff distance, approximately 3σ. The carbon–carbon interactions in the graphene membrane and the CNT were governed by the adaptive intermolecular reactive empirical bond order (AIREBO) potential, while the interactions between carbon and argon were determined using the Lorentz–Berthelot (LB) mixing rule. All the interaction parameters are summarized in Table 1.

**TABLE 1:** Interaction parameters utilized in the simulations.

Interaction	ε (eV)	σ (nm)
Ar –Ar	0.0103	0.3405
C –Ar	0.005	0.3403

Initially, 3900 argon atoms were placed in the two reservoirs of each system (evenly split on both sides) and assigned initial velocities from a Maxwell–Boltzmann distribution at 100 K. The baths were prepared as liquid argon with a bulk‐reservoir number density of ≈ 0.8 to ensure realistic thermodynamic conditions. Temperature control used a Nose–Hoover thermostat, and the system was equilibrated in NVT with a 1 fs timestep: first, a 50 ns equilibration to establish a stable temperature distribution; second, a 100 ns production run in which pressure‐driven flow was generated by translating specular‐reflective walls (SRW) at both the feed and permeate ends at a constant speed of 0.03 m s^−^^1^. Particles colliding with these moving walls underwent specular reflection, so the wall motion compressed one reservoir and expanded the other, producing a steady pressure difference (ΔP) across the constriction and driving argon through the nanopore or CNT without parasitic wall‐fluid interactions. This SRW‐at‐both‐ends pressure‐drive is widely used in nanofluidic MD of graphene nanopores and CNTs [27, 29, 32, 41, 42, 43, 44]. The chosen driving magnitude keeps the system in the linear‐response regime; ΔP rescales the flux while the velocity‐profile shape [27, 45], and thus the inferred resistance trend with wettability and confinement, remains governed by interfacial friction, allowing clear observation of steady single‐file transport.

The pressure tensor of the confined liquid argon was computed by evaluating the normal stresses in the *x*, *y*, and *z* directions, denoted as S_xx_, S_yy,_ and S_zz_, respectively. These were derived using Irving–Kirkwood (IK) formalism [46, 47], which provides a molecular‐scale definition of local stress in inhomogeneous systems. In this approach, the instantaneous pressure tensor for a system of N particles is expressed as

(2) $$S_{\alpha \beta }=\frac{1}{Vol}\langle \sum_{i}m^{i}\left(v_{\alpha }^{i}-u_{\alpha }\right)\left(v_{\beta }^{i}-u_{\beta }\right)+\frac{1}{2}\sum_{i,j}\left(r_{\alpha }^{j}-r_{\alpha }^{i}\right)f_{\beta }^{i,j}\rangle$$

The first term accounts for the kinetic contribution to the stress, where *m^i^* is the mass of particle *i*, *v^i^* is its velocity, and *u* is the streaming velocity in the respective Cartesian direction. The second term represents the virial (configurational) contribution, involving the interatomic force $f_{\beta }^{i,j}$​ and the vector displacement between particles *i* and *j* in the α‐direction. The expression allows stress to be resolved locally within the system and is particularly useful for analyzing confined molecular flows where spatial inhomogeneities are significant. All simulations were performed using the LAMMPS (Large‐scale Atomic/Molecular Massively Parallel Simulator) software, and the results were analyzed using standard MD post‐processing techniques.

Velocity profiles and derived quantities are obtained as long‐time averages under steady conditions (neutral walls; physisorptive LJ interactions; constant state variables). In canonical MD, such time averages are used as consistent estimators of ensemble averages for liquid observables provided the dynamics are stationary and mixing is finite, conditions standardly assumed and documented in the simulation literature [48]. In nanofluidics, steady interfacial‐friction‐dominated flow is routinely characterized from time‐averaged profiles at the sub‐10 nm scale [8]. Our model intentionally excludes non‐stationary driving (e.g., abrupt pressure jumps) and reactive/chemisorptive wall binding; in those scenarios, time–ensemble equivalence may fail, and a non‐ergodic treatment would be required. The conclusions reported here therefore pertain to steady, neutral‐wall confinement, which is the regime targeted in this study.

## 3 Results and Discussion

### 3.1 Number Density Variations and Single‐File Structuring under Confinement

**FIGURE 2:**
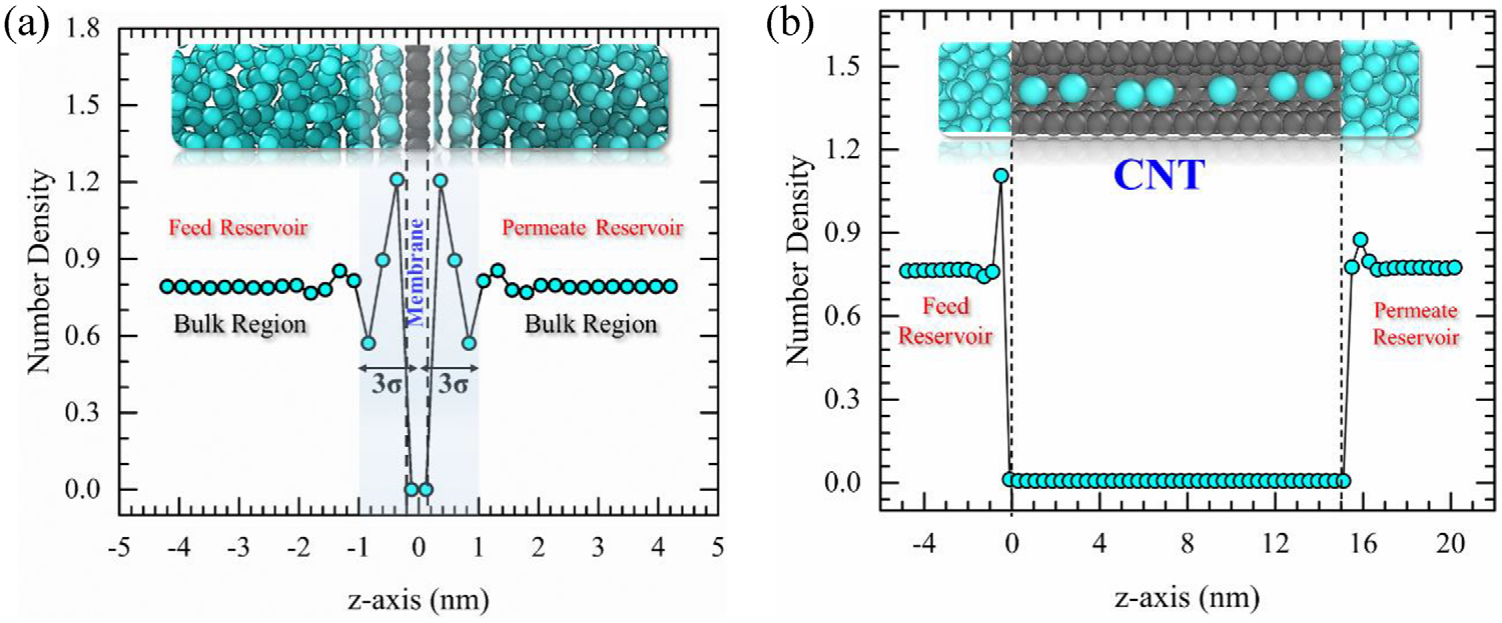
Confinement geometry dictates number‐density structuring. (a) Along the *z*‐axis, the number density for the graphene nanopore shows symmetric layering near the pore (≈ −1 to 1 nm). (b) In the CNT, a pronounced depletion forms inside the tube (0–15 nm) despite single‐file occupancy, with sharp entrance/exit peaks that signal entry resistance and reduced accessible volume under 360° radial confinement. Densities are obtained from 1 Å axial slabs averaged over a 10 ns window; bulk reservoirs maintain ≈ 0.8, confirming the intended thermodynamic state.

Analysis of the number density distributions reveals striking differences between planar nanopores and cylindrical CNTs (Figure 2). In the graphene nanopore, the density layering occurs symmetrically near the pore, indicating strong wall‐fluid interactions that induce alternating high‐ and low‐density regions. In contrast, the CNT exhibits pronounced depletion in the confined region despite single‐file occupancy, with sharp density peaks at the entrance and the exit. This asymmetry indicates directional flow resistance and highlights how cylindrical radial confinement amplifies wall‐fluid interactions, effectively reducing the accessible volume for transport. These findings establish that nanoscale geometry and interfacial forces dictate molecular structuring in ways that are not captured by extrapolating the conventional extrapolation approach of molecular models.

In nanofluidic investigations, it is crucial to analyze the number density distribution of confined fluids because it shows how molecular structure and interaction strength close to interfaces impact transport behavior, particularly under confinement. The radial distribution function, which measures the likelihood of discovering a neighboring particle at a given distance from a reference particle in comparison to an ideal gas, is a key tool for interpreting such molecular configurations [49, 50]. It illustrates how local structuring differs from uniformity and provides information about interatomic forces that drive short‐range ordering. Figure 2a,b shows the number density distribution of liquid argon along the flow direction for two configurations: a nanoporous graphene membrane and a carbon nanotube (CNT), respectively. The simulation domain was divided into slab bins (1Å width) along the axial direction, and density was averaged over a 10 ns equilibrium period. For both systems, the bulk regions exhibit a number density close to 0.8, confirming the correct thermodynamic state of liquid argon. In Figure 2a, corresponding to the nanoporous membrane, symmetric density oscillations are observed near the membrane region (approximately −1 to 1 nm), a phenomenon widely known as density layering [51]. This layering arises from the strong fluid‐solid interactions near the pore entrance, leading to alternating zones of higher and lower atomic packing, a behavior closely tied to radial structuring as characterized by the radial distribution function.

In contrast, Figure 2b presents the density distribution for the CNT case, where the bulk regions maintain a stable number density (∼0.8), similar to the nanoporous membrane configuration. However, a significant depletion of argon atoms is observed inside the CNT (0 to 4 nm). This depletion arises from the narrow diameter of the CNT, which restricts the passage of atoms such that only a single atom can enter at a time, forming a chain‐like structure. Moreover, due to strong wall‐fluid interactions, the effectively accessible volume inside the CNT becomes much smaller than the geometric volume. As a result, the confined region exhibits a substantially lower number density. Also, very sharp density peaks at the CNT entrance and exit further indicate strong confinement effects and potential entry resistance. Notably, an asymmetry appears between the feed and permeate sides in the CNT system, suggesting directional flow resistance. These findings collectively demonstrate how fluid structuring is governed by nanoscale geometry and interfacial forces, which are also reflected in the radial distribution function. This has obvious consequences for membrane performance and design in molecular‐scale separation and flow applications.

### 3.2 Pressure Distribution and Entry Resistance

**FIGURE 3:**
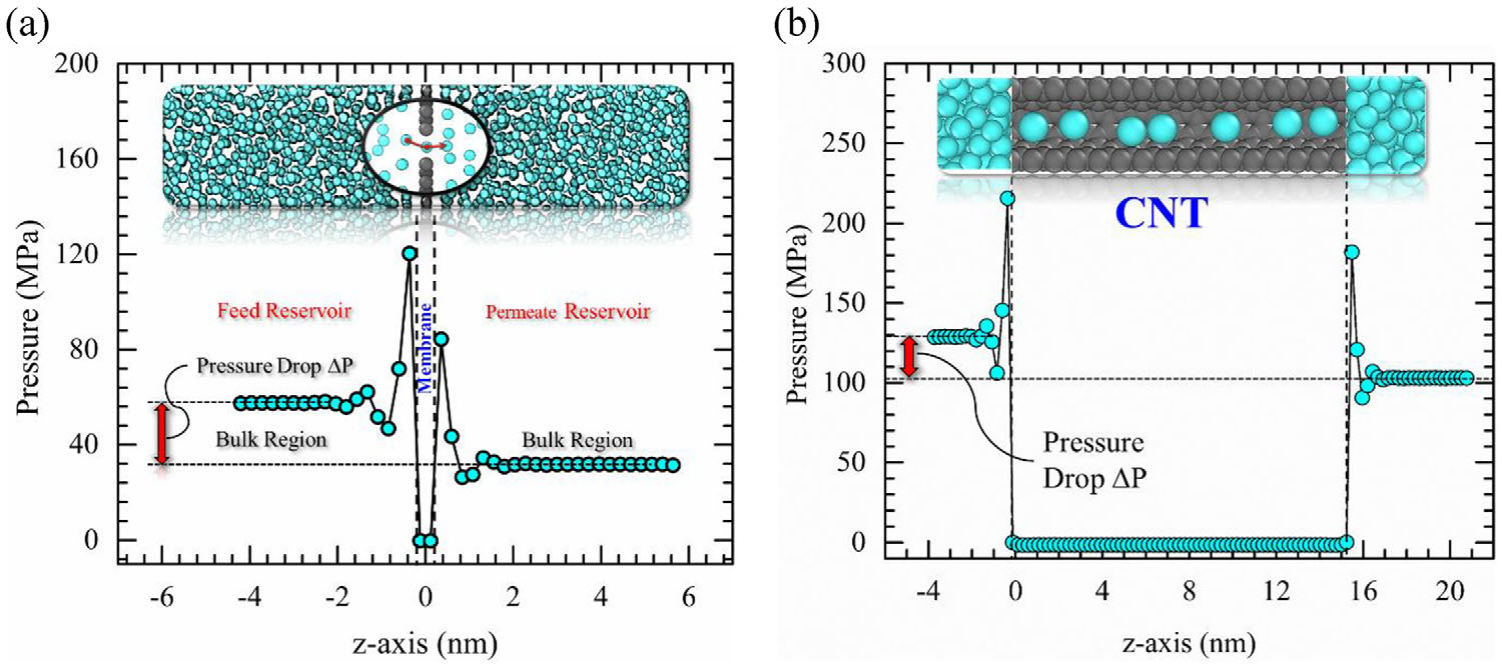
Pressure‐drop patterns reveal where resistance is paid in ultra‐confined flow. (a) The nanopore exhibits a sharp, symmetric ΔP across the 7.45 Å orifice, consistent with single‐file transport dominated by discrete layering at the faces. (b) The CNT shows a smoother overall decline but a higher entrance peak, reflecting the axial alignment required to enter a radially confined channel and the associated local energy barrier. In both geometries, pressure is generated by moving specular‐reflection walls and dissipates predominantly near the exits under steady‐state conditions.

Pressure profiles (Figure 3) further reveal non‐intuitive contrasts between the two geometries. The nanopore exhibits a sharp, symmetric pressure drop across the confined region, consistent with single‐file transport dominated by discrete layering near the walls. The CNT, despite also supporting single‐file transport, shows a more gradual pressure decrease but a higher entrance peak due to 360° radial confinement. This elevated peak arises from the need for atoms to align before entry, creating localized energy barriers that are absent in planar pores. Importantly, the pressure dissipation occurs predominantly at the exit for both systems, facilitating steady‐state transport. These observations emphasize that holistic geometric artefacts and not merely pore diameter control the distribution of flow resistance in ultra‐confined channels.

The detailed pressure‐distribution profiles of the nanopore and CNT systems (Figure 3) further highlight how confinement geometry dictates flow resistance. In both configurations, the pressure difference between the feed and permeate reservoirs originates from the motion of the specular‐reflection walls (SRW), which drive a continuous flux of argon atoms through the constriction. In the nanopore (Figure 3a), the sharp pressure drop across the 7.45 Å orifice signals significant resistance to flow, a direct outcome of extreme confinement where only one atom can occupy the passage at any time. Symmetric layering near the pore entrance and exit confirms strong wall–fluid coupling, arising from the attractive interactions between argon and carbon atoms that produce discrete atomic arrangements. Despite the steep gradient, the overall symmetry of the pressure profile indicates balanced flow resistance on both sides, a hallmark of steady‐state single‐atom transport.

By contrast, the CNT system (Figure 3b) displays a smoother overall pressure decline yet a more pronounced entrance peak. The cylindrical geometry encloses atoms along all directions, strengthening wall‐fluid interactions and generating localized crowding at the inlet. As a result, atoms must align axially to enter the channel, producing a higher pressure barrier than in the planar nanopore. The feed and permeate reservoirs maintain nearly uniform pressures consistent with bulk behavior, while the main resistance is localized inside the CNT. The pressure peaks at the exits of both systems are lower than those at the inlets, reflecting energy dissipation as atoms transition from the confined region back into the reservoirs. This gradient relaxation ensures continuous, steady‐state flow.

Overall, these findings show that confinement geometry, not just pore diameter, sets the spatial distribution of resistance in ultra‐narrow channels: planar pores dissipate energy symmetrically across their faces, whereas cylindrical pores localize it near the entrance due to the requirement of atomic alignment within a radially constrained volume.

### 3.3 Ergodic Recovery of Velocity Profiles

**FIGURE 4:**
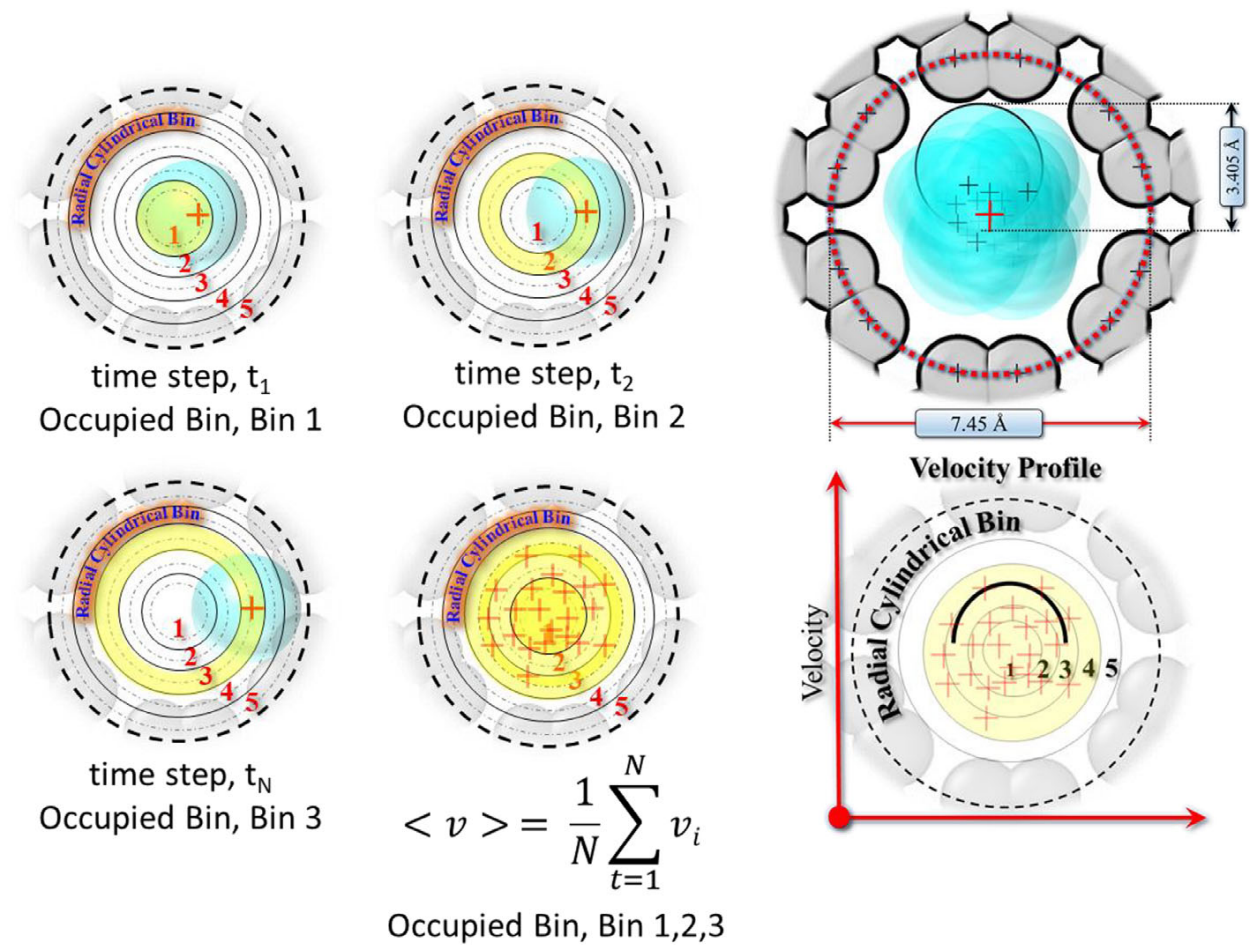
Method of calculating single‐atom velocity profile inside the nanopore. A 7.45 Å graphene pore is partitioned into several concentric radial bins. At each timestep, the single argon atom's axial velocity is assigned to the bin it occupies; long trajectories time‐average these samples to recover a radial velocity profile. This yields a meaningful profile without ensemble averaging or imposing a parabolic shape.

For single‐file transport, ensemble averaging fails with minimal molecular occupancy. By invoking the ergodic hypothesis, we reconstruct statistically meaningful velocity profiles from long‐time trajectories of individual atoms (Figure 4). This time‐averaged approach captures axial velocities across radial bins without assuming parabolic velocity profile extrapolation, providing a bridge between atomistic motion and continuum‐level descriptors.

A central question is whether classical continuum assumptions, especially those tied to viscosity and velocity distributions, remain meaningful when molecular discreteness dominates. In a 7.45 Å nanopore, only one argon atom occupies the passage at a time, so the usual ensemble averaging (many particles simultaneously sampling all spatial bins) is inapplicable. The ergodic hypothesis allows time averages to stand in for ensemble averages in steady systems: given sufficient time, a single particle explores accessible microstates with the same statistical weighting as an ensemble snapshot.

Accordingly, in Figure 4 we illustrate the procedure by dividing the pore cross‐section into concentric radial bins. At each MD step, the atom's instantaneous position and axial velocity are recorded and accumulated in the bin it occupies. Over long trajectories, thermal motion and wall interactions cause repeated visits to each bin. The bin‐wise mean velocity is then computed as $\left\langle v\right\rangle =\frac{1}{N}\;\sum_{t=1}^{N}v_{i}$, yielding a radial velocity profile that approximates the ensemble behavior one would expect from many atoms flowing simultaneously. The schematic shows five bins for clarity; the actual number of bins was chosen case‐by‐case based on pore size and sampling requirements.

This ergodic time‐sampling recovers statistically robust profiles under ultra‐confinement, precisely where fluctuations are large and continuum assumptions break down. It provides a consistent, physics‐based route to map single‐atom trajectories onto continuum‐style descriptors, enabling fair comparison to classical templates without imposing them a priori. This reconstruction strategy is consistent with our recent reports, which document pseudo‐continuum velocity shapes and bin‐wise profile comparisons in sub‐1 nm CNTs and channels, underscoring that this analysis pathway is already recognized by the scientific community [45, 52, 53].

### 3.4 Wettability‐Dependent Flow Behavior and Nonlinear Response

**FIGURE 5:**
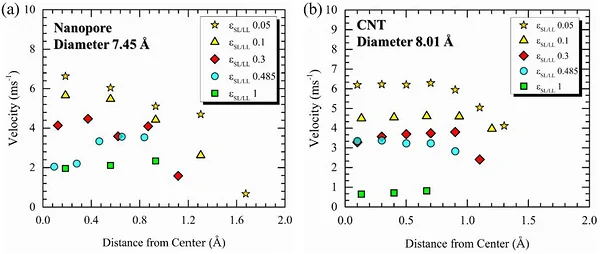
Wettability controls velocity‐profile shape and slip in single‐file flow. (a) Nanopore (radius 3.725 Å): decreasing wettability (ε_SL/LL_ ≈ 0.1–0.05) produces near‐parabolic profiles (Sampson‐like), whereas high wettability (ε_SL/LL_ ≈ 1.0) flattens the profile, indicating stronger interfacial friction and reduced mobility; the intrinsic interaction is ε_SL/LL_ ≈ 0.485. (b) CNT (radius 4 Å): profiles are smoother and more plug‐like across conditions, with consistently larger slip than the nanopore owing to the homogeneous graphitic wall.

The velocity profiles reveal a non‐intuitive dependence on wall‐fluid interactions (Figure 5). In the nanopore, lower wettability (ε_SL/LL_≈ 0.1–0.05) produces nearly parabolic profiles resembling classical predictions, whereas higher wettability (ε_SL/LL_≈ 1.0) flattens the profile, reducing mobility and increasing interfacial resistance. The CNT exhibits smoother, plug‐like flow across wettability values, with lower friction and higher slip velocities due to the homogeneous, radially symmetric wall. Critically, CNTs amplify the effect of wettability: small increases in ε_SL/LL_ dramatically enhance resistance, highlighting a nonlinear sensitivity not seen in planar pores. These findings demonstrate how interfacial friction governs fluidic transport under extreme confinement.

As shown in Figure 5a, the velocity distribution in the nanopore (radius 3.725 Å) is highly sensitive to the solid–liquid interaction ratio (ε_*SL*/*LL*_). We controlled wettability by scaling the solid–liquid Lennard–Jones well depth (ε_SL_) for carbon‐fluid pairs, an approach established for graphite/graphene and CNT interfaces to match or sweep contact angle and, for nanopores, by using simple edge functionalization (H/OH) to modulate local polarity; both practices are standard in MD studies linking wettability to slip and interfacial friction on graphitic surfaces and in nanoporous graphene membranes [33, 54, 55, 56]. At the experimentally representative wettability (ε_*SL*/*LL*_ = 0.485), the velocity profile appears almost linear, although fluctuations are observed due to the limited number of atoms and statistical noise inherent to the confined region. These fluctuations are expected to diminish with longer simulation times or enhanced ensemble averaging.

Interestingly, as wettability decreases (e.g., ε_*SL*/*LL*_ = 0.1 and 0.05), the velocity profile gradually transitions to a parabolic shape, closely resembling the prediction from Sampson's continuum theory for viscous flow through narrow pores. In contrast, higher wettability (ε_*SL*/*LL*_ = 1.0) flattens the profile further, indicating reduced atomic mobility and enhanced wall‐fluid interactions, which dampen near‐wall velocities.

In the CNT case (radius 4 Å), shown in Figure 5b, the velocity profiles are significantly smoother across all ε_*SL*/*LL*_ ratios, owing to the radially symmetric geometry that promotes uniform confinement. At ε_*SL*/*LL*_ = 0.485, the velocity profile is nearly flat, consistent with a plug‐like flow regime indicative of weak wall friction in a single‐file configuration. As wettability decreases, the profile transitions toward a parabolic distribution, closely matching the Hagen–Poiseuille (HP) continuum flow prediction. At high wettability, the velocity significantly decreases due to stronger adhesion of the fluid to the CNT walls.

In contrast, the CNT geometry inherently stabilizes flow and produces clearer trends in velocity distribution. The concept of slip velocity provides further insight into the flow behavior under nanoconfinement. Slip velocity, defined as the fluid velocity at the solid interface, quantifies how freely atoms move along the wall without adhering. In both nanopore and CNT systems, decreasing the ε_*SL*/*LL*_ ratio (weaker wettability) leads to increased slip velocity, as wall‐fluid interactions weaken, allowing atoms to retain more momentum near the wall. However, the CNT consistently exhibits higher slip velocities across all ε_*SL*/*LL*_ratios compared to the nanopore. This is attributed to the atomically smooth and graphitic surface of the CNT, which minimizes friction and promotes plug‐like flow. In contrast, the nanopore's planar confinement introduces more surface disorder and heterogeneous interactions, increasing wall drag and reducing the slip.

The slip velocity at the wall provides a direct measure of interfacial momentum exchange. Across both nanopore and CNT, reducing ε_SL/LL_ (weaker wettability) increases slip as wall‐fluid attraction weakens. The CNT consistently exhibits larger slip because its inner wall presents a nearly homogeneous basal π‐carbon potential with weak lateral corrugation and finite curvature, which together shorten near‐wall residence and lower interfacial friction. In contrast, a graphene nanopore exposes edge (rim) carbons that are under‐coordinated and locally more attractive/rough; combined with orifice‐type entrance/exit dissipation, this increases near‐wall contact and enhances friction, yielding smaller slip for the same ε_SL/LL_. These results are consistent with prior studies [54, 57, 58]. Consequently, wettability modulates resistance more steeply in the nanopore: a larger fraction of the single‐file pathway is forced into the high‐friction first layer near the rim, whereas the CNT maintains a lower‐friction interface over the same ε_SL/LL_ range.

Notably, due to the discrete nature of carbon‐based materials, constructing nanopores or CNTs with arbitrary diameters is not feasible. As a result, wettability becomes an effective control parameter to modulate the effective flow characteristics and emulate continuum‐like behavior in nanoscale systems. This approach allows researchers to bridge the gap between molecular and continuum scales by tuning interfacial interactions instead of geometric dimensions.

### 3.5 Corresponding Viscosity Coefficient as a Molecular Proxy

**FIGURE 6:**
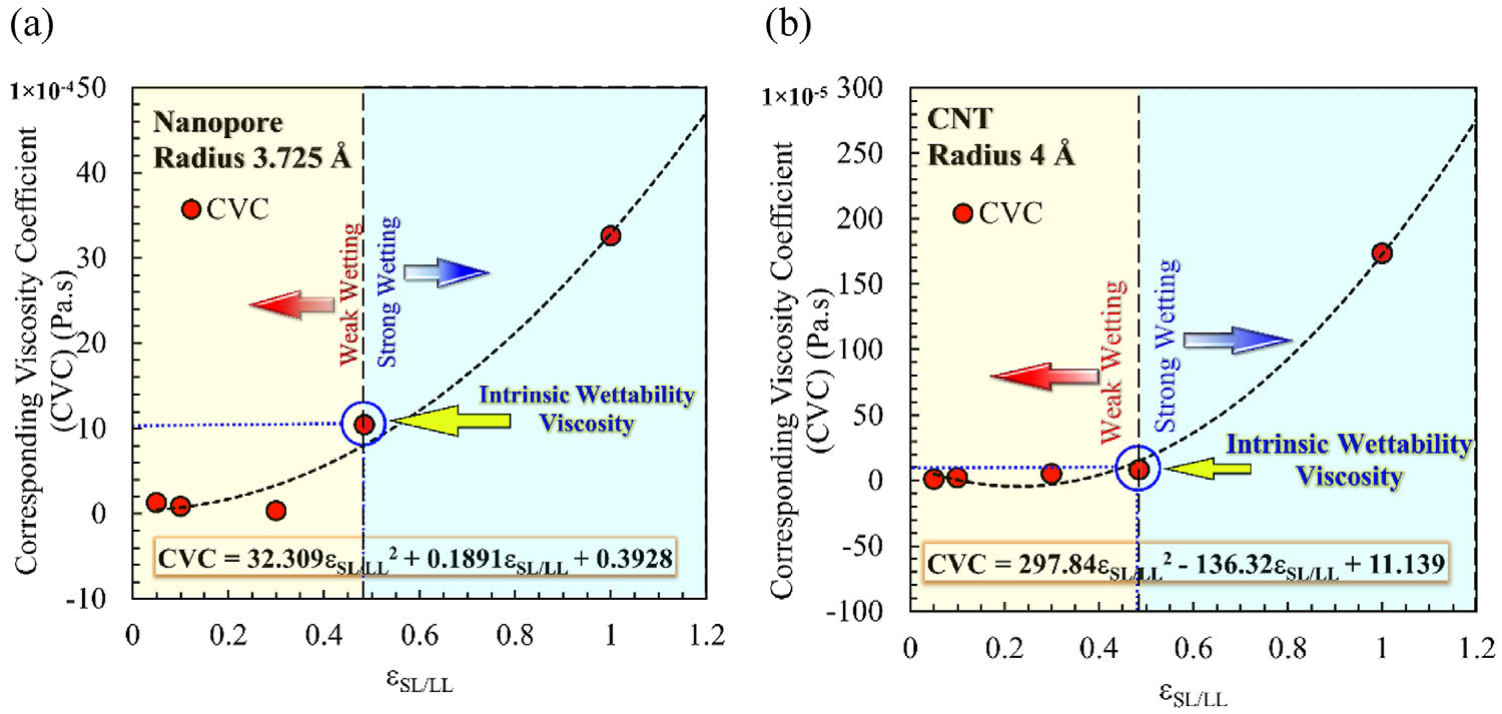
Corresponding viscosity coefficient (CVC) as a function of wettability ε_SL/LL_ for (a) a nanopore with radius 3.725 Å and (b) a CNT with radius 4 Å. Both systems exhibit a quadratic increase in CVC with ε_SL/LL_. The intrinsic wettability point (ε_SL/LL_ = 0.485), corresponding to the natural wall‐fluid interaction strength, is marked in each plot.

We introduce a corresponding viscosity coefficient (CVC) to map MD‐derived velocity profiles to continuum templates (Sampson for pores, Hagen–Poiseuille for tubes) (Figure 6) [45]. In the nanopore, CVC rises quadratically with increasing ε_SL/LL_ beyond the intrinsic interaction (∼0.485), marking a transition from near‐ballistic to interaction‐dominated flow. In the CNT, the rise is even steeper, reflecting the amplified impact of radial confinement on interfacial friction. These trends reveal that molecular resistance emerges here from exclusive atom‐wall interactions, and that tuning wettability can induce unique flow control in such channels.

In Hagen–Poiseuille/Sampson relations, viscosity appears precisely as the coefficient multiplying the profile curvature and throughput [59, 60, 61]. The bulk viscosity is not assumed to hold under sub‐1 nm confinement. Instead, a time‐averaged (ergodic) MD profile is obtained without asserting parabolicity, and the continuum expression is used only as a reference form: the viscosity in that expression is replaced by CVC and chosen so that the template reproduces the MD curvature/throughput for the same confinements (equivalently, viscosity is treated as a fitting coefficient and its counterpart under single‐file conditions is CVC).

It is important to distinguish this procedure from conventional apparent‐ or effective‐viscosity descriptions used in nanoconfined flows. In many continuum‐based corrections, confinement effects are represented by assigning a modified viscosity, slip length, or interfacial friction coefficient to an otherwise continuum‐like flow field [8, 54, 57, 58, 62]. Such approaches are useful when the confined liquid still supports a sufficiently continuous velocity field; however, their interpretation becomes ambiguous in the single‐file regime, where the cross‐section is not continuously occupied, and a conventional local viscosity cannot be defined in the usual sense. The CVC is therefore introduced not as a replacement material viscosity, but as a continuum‐referenced molecular resistance coefficient. Depending on confinement geometry and wall‐fluid interaction strength, the reconstructed MD profiles may appear plug‐like, scattered, flattened, or locally near‐parabolic. The second‐order polynomial is used only as a minimal curvature‐based representation for extracting a comparable resistance coefficient from these profiles, rather than as an imposed continuum velocity field. In this sense, Sampson's and Hagen–Poiseuille relations serve as reference templates for projection of the atomistic profile, not as governing hydrodynamic laws for the single‐file regime.

By construction, CVC is not a bulk material property; it is a configuration‐dependent summary of interfacial, confinement‐dominated resistance tied to the specified geometry, wall‐fluid interaction strength, and steady, neutral‐wall conditions. This definition also provides a natural route for extending the framework beyond the present LJ argon system. In the present study, liquid argon serves as a chemically minimal baseline in which the CVC primarily reflects confinement geometry, discrete occupancy, and wall‐fluid friction. For polar liquids or electrolytes, the same CVC extraction procedure can be applied to the corresponding molecular model, where hydrogen bonding, molecular orientation, hydration structure, electrostatic coupling, and ion‐specific wall interactions can enter through their influence on the reconstructed velocity profile and throughput. Thus, the generality of CVC lies in the mapping framework, while each fluid/surface system yields its own physically meaningful resistance coefficient. Within this definition, the rigid‐wall model should be regarded as part of the specified interfacial configuration. Wall flexibility may quantitatively modify the absolute CVC value by changing interfacial momentum transfer, as suggested by previous studies on CNT‐confined transport [34]. However, because the same rigid‐wall model is applied consistently across all cases, the relative CVC trends with geometry and wall‐fluid interaction strength remain internally comparable within the present controlled framework.

Figure 6 presents the variation of the corresponding viscosity coefficient as a function of solid–liquid interaction strength ε_SL/LL_ for two ultra‐confined nanofluidic systems: a nanopore with a radius of 3.725 Å and a carbon nanotube (CNT) with a radius of 4 Å. In this study, the corresponding viscosity coefficient was calculated by first extracting the velocity profiles of confined argon atoms from molecular dynamics (MD) simulations and then comparing them to classical continuum models. For the nanopore, the radial velocity distribution was fitted to a quadratic curve using the least‐squares method and then aligned with Sampson's analytical equation for pressure‐driven flow through narrow cylindrical pores. This alignment allowed us to relate the curvature of the fitted profile to an effective viscosity, thus defining a corresponding viscosity coefficient that reflects resistance in the single‐file transport regime.

A similar methodology was used for the CNT, where the velocity profile was again fitted to a quadratic form and compared with the Hagen‐Poiseuille (HP) equation, a classical model for viscous flow in cylindrical tubes. The fitting parameters were used to infer corresponding viscosity coefficients under various wettability conditions. In both cases, the approach provides a framework to translate atomistic flow behavior into continuum‐like resistance metrics, even when conventional definitions of viscosity break down. The complete methodology, including velocity profile fitting and curvature matching with Sampson's and Hagen–Poiseuille models, is detailed in Section S3.

In Figure 6a, a clear quadratic rise in CVC is observed with increasing ε_SL/LL_, indicating that as wall‐fluid interactions strengthen, the flow resistance increases substantially. At low ε_SL/LL_ corresponding to low wettability, the corresponding viscosity coefficient remains low and increases only slightly. This behavior reflects weak wall drag, allowing argon atoms to traverse the nanopore with minimal resistance in a plug‐like or ballistic manner. In this regime, the atoms maintain high mobility with limited momentum exchange at the wall, and energy dissipation due to friction is negligible. However, as ε_SL/LL_ increases beyond the intrinsic value of ∼0.485, the corresponding viscosity coefficient rises steeply. This trend signals enhanced atomic interactions with the wall, resulting in decreased slip velocity and a shift from plug‐like to more parabolic velocity profiles, though still far from classical behavior. Importantly, this increase in CVC is not a result of bulk fluid viscosity but instead emerges from the interatomic friction and wall‐molecule adhesion, underscoring the molecular origin of resistance under extreme confinement.

Figure 6b for the CNT system shows a similar qualitative trend but with a much steeper nonlinear increase in CVC. The quadratic fit reveals that flow resistance in the CNT is far more sensitive to wettability than in the planar nanopore. This pronounced behavior arises from the radial confinement geometry of the CNT, where the confined atom is surrounded by carbon walls along 360°, magnifying the impact of wall‐fluid interactions. At low ε_SL/LL_, the corresponding viscosity coefficient remains near zero, indicating nearly frictionless transport with large slip, a hallmark of high‐confinement, low‐wettability environments. However, as ε_SL/LL_ increases, the atoms interact more persistently with the CNT walls, leading to higher resistance, energy dissipation, and a corresponding rise in CVC. The presence of such a strong curvature further highlights that in cylindrical geometries, wettability modulation has an amplified effect on flow behavior.

In both systems, the vertical dashed line at ε_SL/LL_ ≈ 0.485 marks the intrinsic wettability, corresponding to the native interaction strength between argon and carbon. This point corresponds to the crossover from weakly to strongly interacting regimes. The corresponding viscosity coefficient at this intrinsic point offers a reference state for comparing artificially modified wettability conditions. The steep rise in CVC beyond this point illustrates how tuning solid–liquid interactions can drastically alter molecular mobility, even in the absence of changes in fluid structure or external driving forces.

These results strongly support our central hypothesis: in ultra‐confined, single‐file transport regimes, flow resistance emerges not from bulk fluid viscosity, but from molecular‐scale interfacial interactions. The corresponding viscosity coefficient derived here serves as a proxy for this resistance, and its dependence on ε_SL/LL_ reflects how nanoscale friction is fundamentally distinct from classical viscous drag. Modulating wettability thus offers a powerful tool to control transport properties in nanofluidic devices, and the conventional concept of viscosity must be reinterpreted when applied to flows at the molecular scale.

Direct measurements at true single‐file scales (< 1 nm) are still a community‐wide frontier, so we establish credibility in three standard ways. First, our mechanisms (wettability‐controlled interfacial friction and the large slip expected on graphitic carbon) are fully consistent with the closest accessible experiments: ultrafast water flow through sub‐2 nm CNT membranes, orders‐of‐magnitude above continuum expectations, and direct single‐nanotube evidence of massive, radius‐dependent slippage in CNTs but not in BNNTs [24, 58, 63]. Second, our simulations are internally robust across replicas, sampling windows, and systematic sweeps of interaction strength and confinement. Third, we state falsifiable predictions for 1–2 nm channels, e.g., a convex increase of apparent (corresponding‐)viscosity with an interfacial‐friction proxy, and a strong slip advantage for graphitic tubes at matched geometry, that can be confronted as metrology advances using atomically precise graphitic capillaries [64]. These elements align with the nanofluidics consensus that, below a few nanometers, interfacial friction, not bulk viscosity, governs transport.

### 3.6 Statistical Robustness of Single‐Atom Velocity Extraction

**FIGURE 7:**
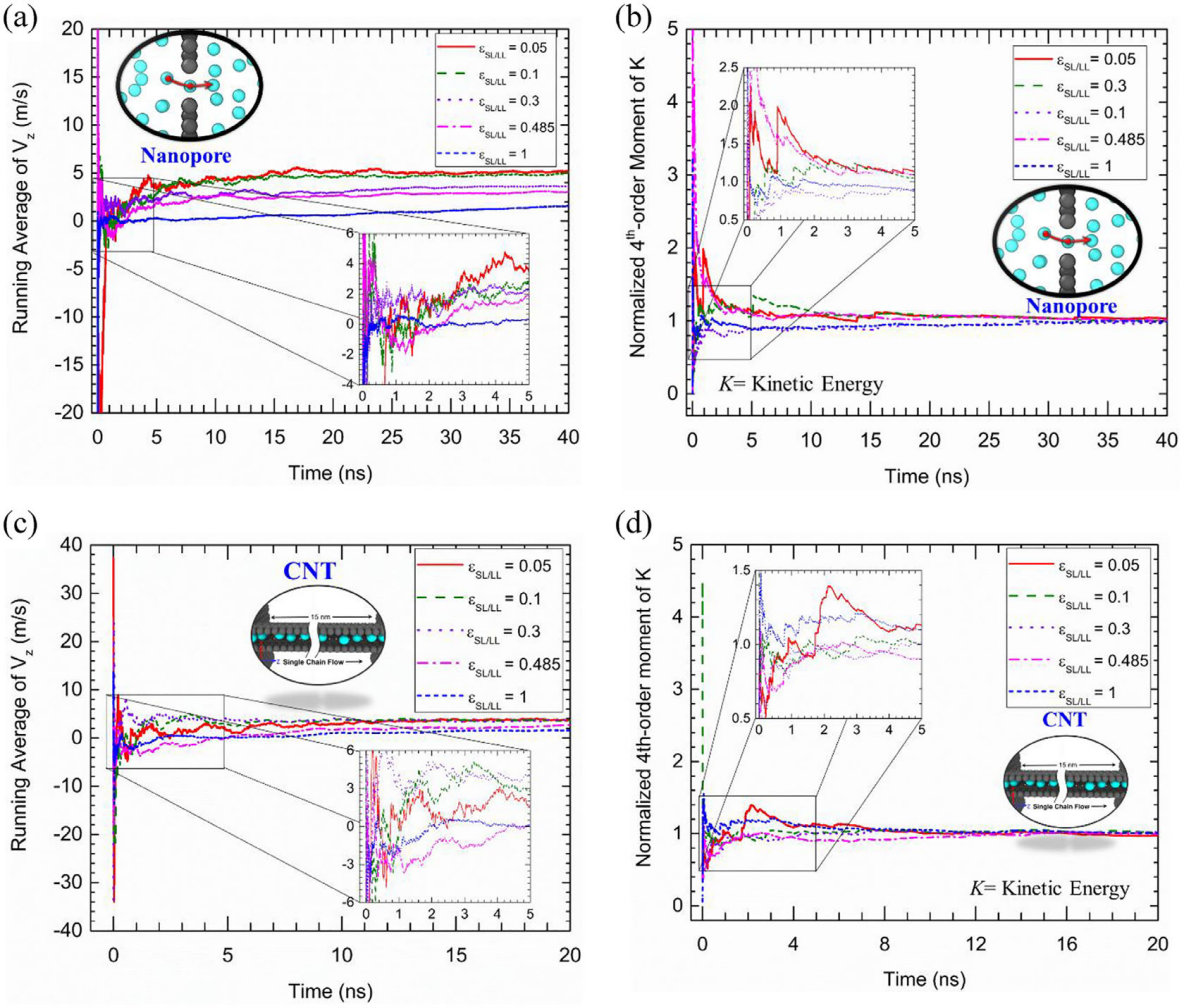
Statistical convergence of axial velocity and kinetic energy fluctuations for nanopore and CNT systems under varying wettability. (a, c) Running average of axial velocity for nanopore and CNT systems, respectively; (b, d) normalized fourth‐order moment of directional kinetic energy for nanopore and CNT systems, respectively.

We validated the ergodic methodology using the running average of axial velocity and the normalized fourth‐order moment of kinetic energy (Figure 7). Nanopores converge more slowly due to intermittent single‐atom occupancy, exhibiting larger high‐energy fluctuations. CNTs form quasi‐continuous single‐file chains, producing faster convergence and reduced non‐Gaussian deviations. These diagnostics confirm that ergodic time‐averaging reliably captures steady‐state transport in ultra‐confined systems and that geometric confinement modulates both resistance and statistical convergence.

To rigorously evaluate the statistical convergence of the confined flow and validate the use of time‐averaged observables in ultra‐confined single‐file systems, we calculated two diagnostic metrics: the running average of axial velocity and the normalized fourth‐order central moment of the directional kinetic energy K. The running average of axial velocity, denoted ⟨v_z_⟩(*t*), is computed as the cumulative average of the z‐component of velocity for atoms located within the membrane region (*z* = ±1.7 nm) up to time *t*. It is defined as:

(3) $$\langle v_{z}\rangle \left(t\right)=\frac{1}{N\left(t\right)}\sum_{i=1}^{N\left(t\right)}v_{z,i},$$

where *v*_*z*,*i*_​ is the instantaneous axial velocity of atom *i*, and *N*(*t*) is the total number of contributing data points up to time *t*. This metric reveals how the axial transport behavior stabilizes over time.

To probe the statistical nature of kinetic energy fluctuations, we also compute the normalized fourth‐order central moment of the directional kinetic energy:

(4) $$K_{i}=\frac{1}{2}mv_{z,i}^{2}$$

where *m* is the mass of the argon atom. The central 4th‐order moment over a time window up to *t* is:

(5) $$\mu_{4}\left(t\right)=\langle {\left(K-\langle K\rangle \right)}^{4}\rangle$$

and its normalized form is given by:

(6) $$\tilde{\mu_{4}}\left(t\right)=\frac{\mu_{4}\left(t\right)}{\mu_{4}\left(t_{max}\right)}$$

where〈*K*〉 is the mean kinetic energy over the same interval and *t_max_* ​ is the total simulation time. This normalized value measures the deviation of the energy distribution from Gaussian behavior and the persistence of high‐order fluctuations over time [65, 66].

Additionally, we validated that the atomic velocity distribution follows Maxwell–Boltzmann statistics, confirming thermal equilibrium and supporting the ergodic assumption (see Section S1). Figure 7a,c presents the running average of v_z_ for the nanopore and CNT, respectively. In both systems, the early‐time signal oscillates because the number of atoms inside the analysis region is small. The nanopore converges more slowly and with larger excursions, since the pore is typically occupied by only one argon atom at a timestep. This intermittent occupancy produces sparse sampling and long correlation times, so more simulation time is required for the cumulative average to stabilize. By contrast, the CNT forms a quasi‐continuous single‐file chain of argon along the axis; multiple atoms reside inside the tube simultaneously, which increases the effective sample size per time step and yields faster, smoother convergence of the running average.

Figure 7b,d shows the normalized fourth‐order central moment of the directional kinetic energy. Both geometries exhibit an initial spike followed by relaxation toward unity as the statistics accumulate. The nanopore displays larger early deviations and a slower decay, consistent with intermittent, high‐energy transits of single atoms and the associated non‐Gaussian tails. The CNT relaxes more rapidly and with smaller fluctuation amplitude, reflecting its cylindrically symmetric confinement, smoother wall landscape, and sustained single‐file occupancy that promotes more ergodic sampling.

Overall, Figure 7 provides statistical validation of our analysis framework. The running average confirms that steady axial transport can be characterized in ultra‐confined flows, but the nanopore requires longer averaging windows than the CNT. The normalized fourth‐order moment further shows that geometry and wettability control not only resistance but also the rate of statistical convergence: systems that maintain a single‐file chain (CNT) stabilize quickly, whereas intermittent single‐atom occupancy (nanopore) demands extended sampling. These diagnostics support the use of ergodicity‐based time averages for velocity extraction in regimes where ensemble averaging is impractical.

To further verify the convergence of the reconstructed velocity profiles, we performed an additional sampling‐duration test for the representative low solid–liquid interaction case (ε_SL/LL_ = 0.1) in both nanopore and CNT geometries, as shown in –Figure S2. The velocity profiles were reconstructed using progressively longer trajectory windows while keeping the same binning and averaging procedure. In the nanopore, the short 20 ns trajectory shows a large deviation, especially near the outer radial bin, and the 50 ns profile still exhibits noticeable edge‐bin fluctuation. With longer sampling windows, however, the profiles approach the same overall shape and magnitude. In contrast, the CNT profiles converge more rapidly, with the 50, 70, and 100 ns profiles nearly overlapping over most radial positions. This difference further supports that the nanopore requires longer averaging due to sparse and intermittent single‐atom occupancy near the pore edge, whereas the CNT provides more sustained axial single‐file occupancy. Since CVC is derived from the fitted long‐time velocity profile rather than sampled as an independent molecular observable, the stability of the reconstructed velocity profile provides the most direct convergence criterion for the CVC values reported in this work.

### 3.7 Key Insights and Implications

The results described above reveal several key insights into the viscous resistances in angstrofluidic transport phenomena. Single‐file molecular motion is found to exhibit a pronounced geometry‐dependent asymmetry in density, pressure, and velocity distributions, fundamentally challenging the paradigm of conventional continuum‐mirroring models. Under cylindrical confinement, interfacial friction is significantly amplified, leading to a nonlinear dependence on surface wettability and underscoring the pivotal influence of wall‐fluid interactions. To reconcile molecular‐scale resistance with continuum descriptions, the corresponding viscosity coefficient (CVC) emerges as a practical and predictive surrogate, facilitating the rational design of sub‐1 nm nanofluidic devices. Furthermore, the validation of ergodic averaging as a reliable means of extracting velocity profiles, particularly where ensemble averaging proves inadequate, establishes a robust methodological bridge between atomistic simulations and device‐level modeling. We emphasize that the present comparison is not intended to constitute a complete parametric geometry map. Rather, it focuses on representative atomistically allowed single‐file confinements: an atomically thin graphene nanopore and a finite‐length cylindrical CNT. Within this controlled comparison, the CVC is strongly modulated by confinement geometry and wall‐fluid interaction strength. A systematic sweep of pore diameter, CNT chirality/diameter, and channel length would be valuable for constructing a generalized angstrofluidic resistance map and is identified as an important direction for future work.

## 4 Conclusion

This study reveals the fundamental origin of fluid resistance in sub‐nanometer single‐file transport, a regime where traditional notions of viscosity and wettability lose their physical relevance. Using molecular dynamics simulations of liquid argon confined within graphene nanopores and carbon nanotubes, we demonstrate that flow resistance arises not from bulk fluid characteristics but from direct molecular interactions with the confining surfaces. These insights challenge the validity of continuum‐based models and underscore the necessity to redefine transport parameters at the molecular scale.

By invoking the ergodic hypothesis, we extract statistically robust velocity profiles from single‐molecule trajectories and introduce a corresponding viscosity coefficient (CVC) to quantify flow resistance under varying wall‐fluid interactions. The CVC exhibits a pronounced nonlinear dependence on wettability, indicating that at the nanoscale, friction is governed predominantly by interfacial physics and confinement geometry rather than by bulk molecular interactions. This formulation provides a tangible bridge between atomistic behavior and continuum‐like representations, enabling predictive modeling of angstrofluidic transport.

The implications of these findings extend across diverse domains involving extreme confinement, such as semiconductor manufacturing, nanofiltration, and molecular sensing, where subcontinuum transport dictates performance and precision. Moreover, by decoupling confinement effects from electrochemical or electromechanical influences, this work establishes a foundational framework for extending such analyses to charged, polar, or functionalized systems. In this progression, the present LJ argon model represents the simplest baseline case, while future SPC/E‐water and electrolyte models can incorporate hydrogen bonding, hydration structure, polarity, and Coulombic interactions within the same CVC‐based analysis framework. It opens the way to the rational design of nanomaterials and devices with tunable flow resistance, emphasizing that at the sub‐nanometer scale, resistance transforms from a bulk property into a controllable interfacial phenomenon governed by geometry, molecular interactions, and statistical mechanics.

## Conflicts of Interest

The authors declare no conflicts of interest.

## Supporting information




**Supporting File**: smll75044‐sup‐0001‐SuppMat.docx.

## Data Availability

The data that support the findings of this study are available from the corresponding author upon reasonable request.
