# Supplementary material for: Molecular Friction Trumps Viscosity in Angstrofluidic Transport
Source: Small. 2026 Aug 4;22(51):e75044. doi: 10.1002/smll.75044 (PMC13568852; doi:10.1002/smll.75044)
Supplement: Supplementary file 1 [file SMLL-22-e75044-s001.docx]

**Supplementary Material**

**Molecular Friction Trumps Viscosity in Angstrofluidic Transport**

Jaber Al Hossain^a^, Murat Barisik^b^, Chirodeep Bakli^c^, Suman Chakraborty^d^ and BoHung Kim^a,*^

^a^ School of Mechanical Engineering, University of Ulsan, Daehak-ro 93, Namgu, Ulsan 680-749, South Korea

^b^ Mechanical Engineering Department, University of Tennessee at Chattanooga, Chattanooga, TN 37403, USA

^c^ School of Energy Science and Engineering, Indian Institute of Technology Kharagpur, Kharagpur, West Bengal, 721302, India-

^d^ Department of Mechanical Engineering, Indian Institute of Technology Kharagpur, Kharagpur, West Bengal, 721302, India

**^*^** Corresponding Author / E-mail: [bohungk@ulsan.ac.kr](mailto:bohungk@ulsan.ac.kr), TEL: +82-52-259-2705

**S1. Validation of thermal equilibrium:**

In classical statistical mechanics, the velocity distribution of atoms in a system at thermal equilibrium follows the Maxwell–Boltzmann statistics. For a single velocity component 𝑣_𝑧_, this reduces to a Gaussian (normal) distribution:

$$f\left( v_{i} \right)=\frac{1}{\sqrt{\frac{2\pi k_{B}T}{m}}}exp\left( -\frac{mv_{i}^{2}}{2k_{B}T} \right)$$

(1)

Where, $v_{i}$is the velocity component, $m$ is the mass of the atom, $k_{B}$ is the Boltzmann constant and $T$ is the temperature. This distribution forms the statistical foundation for equilibrium thermodynamics in molecular dynamics (MD) simulations. Verifying that the velocity components of atoms follow this distribution is essential for confirming that the system has reached equilibrium, particularly in nanoscale systems where conventional ensemble averaging is limited due to small atom counts.

In our study, we validated the equilibrium state of the simulation by calculating the probability density function of 𝑣_𝑧_ for liquid argon atoms and fitting it to the Gaussian function above. This analysis was performed for two cases: (a) the entire simulation domain and (b) only for atoms confined within the carbon nanotube (CNT) channel (5 Å < z < 145 Å). The results, shown in Figure S1, exhibit strong agreement between MD simulation data and the theoretical Gaussian fit in both domains. This confirms that the atomic motions obey classical thermal statistics, thereby validating the ergodic assumption and enabling the use of long-time averages for further transport analysis. This step is crucial to the broader aim of our work. Since single-file transport prohibits conventional ensemble-based velocity profiling due to the presence of only one atom in the channel at a time, we rely on time-averaged trajectories of individual atoms. Verifying that these trajectories produce velocity distributions consistent with Gaussian statistics ensures that our velocity reconstruction methodology is statistically sound and that the system can be meaningfully compared to continuum concepts, although it relies on time-averaged data instead of ensemble averages.

| (a) | (b) |
| --- | --- |
|  |  |

Figure S1. Gaussian distribution of the axial velocity component 𝑣_z_ of argon atoms at equilibrium.

(a) Velocity distribution for the entire simulation domain. (b) Velocity distribution restricted to argon atoms located inside the CNT channel only (5 Å < z < 145 Å). In both cases, the histograms represent molecular dynamics (MD) simulation data, while the dashed lines show the Gaussian fit based on the Maxwell–Boltzmann distribution for a single velocity component. The strong agreement confirms that the system is well-equilibrated and follows classical thermal statistics.

**S2. Sampling-duration convergence of ergodic velocity reconstruction**

To directly verify the statistical convergence of the ergodically reconstructed velocity profiles, we performed a sampling-duration test for the representative low solid-liquid interaction case, ε_SL/LL_=0.1, in both nanopore and CNT geometries. The radial velocity profiles were reconstructed using progressively longer trajectory windows while keeping the same binning and averaging procedure used in the main analysis.

| (a) | (b) |
| --- | --- |
|  |  |

Figure S2. Sampling-duration convergence of ergodically reconstructed velocity profiles at ε_SL/LL_=0.1; (a) Nanopore case and (b) CNT case. Short sampling windows, especially 20 ns, show visible statistical deviations. With increasing sampling duration, the reconstructed profiles approach stable long-time trends. The nanopore requires longer averaging because of sparse and intermittent single-atom occupancy near the pore edge, whereas the CNT converges more rapidly due to sustained axial single-file occupancy inside the cylindrical confinement.

Figure S2(a) shows the nanopore case. The short 20 ns trajectory exhibits a large deviation, especially near the outer radial bin, indicating that short sampling is insufficient for stable velocity reconstruction. The 50 ns profile improves but still shows noticeable edge-bin fluctuation. With longer sampling windows of 70, 80, 90, and 100 ns, the velocity profiles approach the same overall shape and magnitude. The remaining scatter near the pore edge is attributed to sparse and intermittent single-atom occupancy in the narrow pore region.

Figure S2(b) shows the CNT case. Compared with the nanopore, the CNT velocity profiles converge more rapidly. The profiles obtained from 50, 70, and 100 ns nearly overlap over most radial positions, while the 20 ns profile still shows visible deviation. This faster convergence is attributed to the extended cylindrical confinement of the CNT, which provides more sustained axial single-file occupancy and better time sampling than the atomically thin nanopore.

Overall, Figure S2 confirms that the velocity profiles used for the main CVC analysis are not determined by short-time fluctuations. The nanopore requires longer averaging because of intermittent occupancy near the pore edge, whereas the CNT reaches stable velocity reconstruction more rapidly.

Since the CVC is not a directly sampled molecular observable but a derived coefficient obtained from the fitted long-time velocity profile, the convergence assessment was performed on the primary quantity entering the CVC calculation, namely the ergodically reconstructed velocity profile. As shown in Figure S2, short sampling windows produce visible deviations, especially for the nanopore edge bins, whereas the longer sampling windows recover stable velocity-profile shapes. Therefore, the CVC values reported in the main manuscript were extracted from the final long-time averaged velocity profiles, for which the primary reconstructed velocity profiles showed statistical stability. We did not interpret short-time fitted CVC values as independent transport coefficients because the curvature-based fitting is highly sensitive to sparsely occupied edge bins under single-file transport.

**S3. Calculation methodology of Corresponding viscosity coefficient (CVC)**

To quantify flow resistance under ultra-confined conditions where conventional fluid viscosity loses its physical meaning, we introduce a corresponding viscosity coefficient (CVC) by aligning molecular dynamics (MD) velocity profiles with classical continuum models. This section details the numerical procedure used for this extraction**.**

**S3.1. Velocity profile fitting from MD data**

For each wettability condition, the radial velocity profile 𝑣(𝑟) of argon atoms inside the nanopore or CNT was extracted via ergodic averaging (see main text, Fig. 4). The simulation domain was divided into radial bins, and the velocity data were fitted to a second-order polynomial:

$$v\left( r \right)= {Ar}^{2}+Br+C$$

(2)

Here, 𝐴, 𝐵, and 𝐶 are fitting coefficients obtained by solving the system of normal equations using least squares regression. The raw data points were drawn from time-averaged molecular positions and velocities, ensuring the reconstructed profile represents long-time flow behavior under confinement.

| (a) | (b) |
| --- | --- |
|  |  |

Figure S3. Quadratic velocity profile fitting for ultra-confined argon flow at wettability ratio SL/LL 0.05. (a) Nanopore with a diameter of 7.45 Å, and (b) carbon nanotube (CNT) with a diameter of 8.01 Å.

An example of this fitting process for the nanopore at 𝜀_𝑆𝐿/𝐿𝐿_=0.05 is shown in Figure S3a, where the final fit was:

$$v\left( r \right)={-3.2616x}^{2}+2.5154x+6.0207$$

(3)

This quadratic form provides a minimal curvature-based representation of the ergodically reconstructed MD profile, enabling coefficient matching with the continuum reference expression without assuming that the molecular velocity profile is strictly parabolic.

**S3.2. Matching with Sampson’s continuum equation**

To extract CVC**,** the fitted velocity profile was compared with Sampson’s analytical solution for viscous flow through a thin circular pore:

$$v\left( r \right)\text{ =}\frac{a\Delta P}{2\pi\mu}\sqrt{1-\left( \frac{r}{R} \right)^{2}}$$

(4)

This was algebraically rearranged into a matching parabolic form:

$$v\left( r \right)\text{ =}k_{1}-k_{2}r^{2}$$

(5)

By aligning the MD-fitted and analytical coefficients (comparing constants and curvature), CVC was extracted:

$$\text{CVC =}\frac{a\Delta P}{2\pi k_{2}}$$

(6)

In the example above, using the matched curvature term and known pressure drop, the corresponding viscosity coefficient was computed as:

$$\mu_{0.05}=1.31307815\times{10}^{-5} pa.s$$

**S3.3. Extension to other cases**

This process was repeated for:

Other wettability ratios and the CNT geometry, where classical Hagen-Poiseuille (HP) equation was used instead of Sampson’s:

$$v\left( r \right)=\frac{\Delta P}{4\mu L}\left( R^{2}-r^{2} \right)$$

(7)

Here, a similar coefficient-matching strategy was applied, equating the curvature of the fitted profile with that from the HP model to compute CVC.
